# Narrative exposure therapy for refugees and asylum seekers with PTSD: A review of the literature

**DOI:** 10.1192/j.eurpsy.2021.1198

**Published:** 2021-08-13

**Authors:** A. Macallin, R. Wynn

**Affiliations:** Department Of Clinical Medicine, UiT The Arctic University of Norway, Tromsø, Norway

**Keywords:** Refugee, Asylum seeker, Narrative Exposure Therapy, ptsd

## Abstract

**Introduction:**

Refugees and asylum seekers have often been exposed to multiple or complex traumas and are known to have a high rate of trauma-related disorders. Different therapeutic approaches have been used to treat this group with varying success. Narrative Exposure Therapy (NET) is one promising intervention for refugees and asylum seekers that are suffering from post-traumatic stress-disorder (PTSD). NET is a treatment given individually or in small groups in typically 12 sessions or less. In NET, memories are reorganized through a process involving imaginary exposure to trauma.

**Objectives:**

To review the literature on NET for refugees and asylum seekers suffering from PTSD.

**Methods:**

The data bases PubMed, Medline, PsycInfo and Web of Science were searched using a selection of search terms, including ‘Narrative therapy’, ‘refugees’ and ‘stress disorders, post-traumatic’. The identified relevant articles were qualitatively assessed and effect sizes were compared. Methodological quality was assessed according to the GRADE-criteria.

**Results:**

Thirteen studies were assessed with a total study population of 745. Nine of the included studies were RCTs. Overall, the studies found medium to very high effects of NET. The quality of the studies varied from very low to high. More studies of NET for refugees and asylum seekers are needed, and in particular studies reporting long-term outcomes.

**Conclusions:**

The review suggests that NET shows promise as a method for the treatment of PTSD in refugees and asylum seekers. However, the review is based on relatively few studies and more studies of long-term outcomes are particularly needed.

